# Somatosensory evoked potentials and magnetic resonance imaging of the central nervous system in early multiple sclerosis

**DOI:** 10.1007/s00415-022-11407-1

**Published:** 2022-10-07

**Authors:** Alexander Wuschek, Matthias Bussas, Malek El Husseini, Laura Harabacz, Viktor Pineker, Viola Pongratz, Achim Berthele, Isabelle Riederer, Claus Zimmer, Bernhard Hemmer, Jan S. Kirschke, Mark Mühlau

**Affiliations:** 1 Department of Neurology, School of Medicine, Technical University of Munich, Ismaninger Str. 22, 81675 Munich, Germany; 2 TUM-Neuroimaging Center, School of Medicine, Technical University of Munich, Munich, Germany; 3 Dept. of Neuroradiology, School of Medicine, Technical University of Munich, Munich, Germany; 4 Munich Cluster for Systems Neurology (SyNergy), Munich, Germany

**Keywords:** MRI, Evoked potentials, Multiple sclerosis

## Abstract

**Background:**

Somatosensory evoked potentials (SSEP) are still broadly used, although not explicitly recommended, for the diagnostic work-up of suspected multiple sclerosis (MS).

**Objective:**

To relate disability, SSEP, and lesions on T2-weighted magnetic resonance imaging (MRI) in patients with early MS.

**Methods:**

In this monocentric retrospective study, we analyzed a cohort of patients with relapsing–remitting MS or clinically isolated syndrome, with a maximum disease duration of two years, as well as with available data on the score at the expanded disability status scale (EDSS), on SSEP, on whole spinal cord (SC) MRI, and on brain MRI.

**Results:**

Complete data of 161 patients were available. Tibial nerve SSEP (tSSEP) were less frequently abnormal than SC MRI (22% vs. 68%, *p* < 0.001). However, higher EDSS scores were significantly associated with abnormal tSSEP (median, 2.0 vs. 1.0; *p* = 0.001) but not with abnormal SC MRI (i.e., at least one lesion; median, 1.5 vs. 1.5; *p* = 0.7). Of the 35 patients with abnormal tSSEP, 32 had lesions on SC MRI, and 2 had corresponding lesions on brain MRI.

**Conclusion:**

Compared to tSSEP, SC MRI is the more sensitive diagnostic biomarker regarding SC involvement. In early MS, lesions as detectable by T2-weighted MRI are the main driver of abnormal tSSEP. However, tSSEP were more closely associated with disability, which is compatible with a potential role of tSSEP as prognostic biomarker in complementation of MRI.

## Introduction

Until magnetic resonance imaging (MRI) entered clinical routine, evoked potentials had been the most important paraclinical tool to objectively detect pathological changes in multiple sclerosis (MS) [1]. Ever since, evoked potentials have remained part of the routine diagnostic work-up in many centers. Yet current diagnostic criteria attribute a role only to visual evoked potentials to lend objective paraclinical evidence in a patient reporting a previous episode of visual impairment whereas the possible contribution of other evoked potential investigations is recommended to be further explored [2]. Somatosensory evoked potentials (SSEP) cover the whole spinocortical pathway; they are robust and easy to perform. According to some authors, SSEP are among the most valuable electrophysiological tests in MS [3] with the highest sensitivity [4] and of prognostic value [5–9]. However, with the broader availability of MRI including spinal cord (SC) MRI, the question has arisen whether SSEP are of value beyond MRI and how the findings of both methods relate to each other. To the best of our knowledge, only very few studies reported findings from both methods [10]. In this study, we retrospectively analyzed disability, SSEP, brain MRI, and whole SC MRI with full axial coverage in a larger cohort of patients with early MS.

## Methods

### Participants

This retrospective analysis was part of the single center cohort study on MS at the Technical University of Munich (TUM-MS), which was approved by the internal review board and performed in accordance with the Declaration of Helsinki. Inclusion criteria were a diagnosis of clinically isolated syndrome (CIS) or relapsing–remitting MS (RRMS), an age between 18 and 60 years, and a disease duration of less than 2 years. To achieve a uniform classification of patients, all patients of the cohort analyzed in this study were reclassified according to the 2017 diagnostic criteria [2]. CIS patients were defined by a first clinical event suggestive of RRMS and fulfilling the criteria for dissemination in space but not fulfilling the criteria for dissemination in time. Further inclusion criteria were availability of a score on the Expanded Disability Status Scale (EDSS), of a standardized quality-checked and processed brain MRI (based on a protocol exclusively used between 2009 and 2017, for details see [11]), and of a SC MRI with coverage from the foramen magnum to the conus medullaris in both the sagittal and axial plane. The maximum interval between either pair of the four measures (EDSS, tSSEP, SC MRI, brain MRI) was set to 200 days; patients with a relapse during this interval were excluded from analysis.

### Somatosensory evoked potentials

SSEP were conducted routinely in the context of the diagnostic workup by stimulating the median nerve at the wrist and the tibial nerve at the ankle, both with a threefold of the sensory perception threshold and a stimulus frequency of 3 Hz. According to the 10/10 system, the recording electrodes were placed at CP3/CP4 (median nerve) and CPz (tibial nerve) with the reference electrode at Fz. Median nerve N20 peak latency and amplitude were measured. Tibial nerve P40 peak latencies were corrected for body height resulting in the unit ms/m. An amplitude of less than 0.6 µV (median nerve) and 1.0 µV (tibial nerve) or a latency of ≥ 22.3 ms (median nerve) or of ≥ 26 ms/m (tibial nerve) on either side was classified as abnormal according to in-house normative values very similar to those (25.7 ms/m as upper limit for tSSEP) reported in [12]. For group comparison, the larger latency value of both sides was used.

### Acquisition and processing of spinal cord MRI

Details on the processing of SC MRI have been reported recently [13]. In short, SC MRI was performed at three 3-Tesla scanners (Philips Achieva dStream, Philips Ingenia, Siemens Magnetom Verio). A spine coil was used and optionally an anterior body coil. All scans included 2D T2-weighted (w) turbo spin echo sequences in sagittal and axial orientation. Sequence parameters were not constant and are given in median (range) according to the order of scanners given above. Sagittal scans had a slice thickness of 2 mm with a gap of 0.2 mm; respective echo times (TE) in ms were 107 (107–111), 120 (100–120), and 107 (107–111); repetition times (TR) in ms were 3000 (3000–3000), 3000 (2800–3393), and 3000 (3000–3000); long echo train length (ETL) in ms were 21, 30 (29–33), and 21; compressed sense (CS) factors were 1.7 (0–2), 1.7 (0–2), and none. Respective fields of view (FOV) in mm were 220 (220–230), 266 (136–579), and 220 (220–320) with in-plane spatial resolutions of 0.57 (0.57–0.83), 0.31 (0.28–0.57), and 0.57 (0.57–0.83). Axial scans were acquired in three consecutive stacks; they had a slice thickness of 4 mm with a gap of 1 mm; TE in ms were 90 (90–110), 100 (90–110), and 107 (105–107); TR in ms were 4531 (3464–5528), 4573 (3521–5528), and 7070 (4120–8764); ETL in ms were 21 (20–21), 21 (20–21), and 19; CS factors were 1.7 (0–2), 1.7 (0–2), and none. FOV in mm were 102 (66–218), 120 (66–218), and 220 (183–264) with in-plane spatial resolutions in mm of 0.23 (0.19–0.34), 0.34 (0.19–0.34); and 0.69 (0.57–0.69). All scans were converted to NIFTI file format and segmented with the software BrainSeg3D, Version 2.2.1 (http://lit.fe.uni-lj.si/tools.php?lang=eng). Lesions were segmented fully manually. Lesion numbers were derived automatically, and volumes of all lesions summed up. SC MRI showing at least one lesion were classified as abnormal.

### Acquisition and processing of brain MRI

Standardized brain MRI was performed at one and the same 3 Tesla scanner Achieva (Philips Medical Systems, Netherlands; 3D spoiled gradient echo T1-weighted sequence with a voxel size of 1 mm isotropic, and TR = 9 ms, TE = 4 ms; turbo-spin echo T2-weighted fluid-attenuated inversion recovery sequence with a voxel size of 1.0 × 1.0 × 1.5 mm, and TR = 10,000 ms, TE = 140 ms, TI = 2750 ms).

### Statistical analysis

Of note, SC lesion volume and tSSEP turned out to be a challenge for statistical analysis. In patients without a single SC lesion, the volume is zero resulting in a distribution across the cohort that is problematic even for rank-based non-parametric tests. Fully absent tSSEP constitute an even bigger challenge as neither P40 latency nor amplitude could be determined. Therefore, we decided to dichotomize the results of both methods (abnormal vs. normal) and to make use of the fact that in all patients with normal tSSEP, a distinct value of the P40 latency for correlation analyses is available. Likewise, all patients with SC lesions have a lesion volume > 0. For clarity, we will refer to the whole of our patients as cohort. Patients defined by dichotomized results of one method are referred to as groups (Table 1) and patients defined by dichotomized results of both methods as subgroups (Table 2). Subgroup 1 has normal results on both methods, subgroup 2 has normal tSSEP but abnormal SC MRI, and subgroup 3 abnormal results on both methods. Frequency distribution of dichotomized findings (normal vs. abnormal) of SC MRI and tSSEP were tested for differences by Fisher’s exact test. Only non-parametric tests were used (Fisher’s exact test for comparison of ratios; Wilcoxon–Mann–Whitney-Test for group comparisons, and Spearman rho for simple and partial correlations). Two-sided *p* values are given.

**Table 1:** Key characteristics of patients

	All	Normal tSSEP	Abnormal tSSEP	No lesions on SC MRI	≥ 1 lesion on SC MRI
*N*	161	126	35	51	110
Female/male	112/49	89/37	23/12	36/15	34/76
CIS/RRMS	18/143	17/109	1/34	13/38	5/105
Disease duration in years	0.061 [0.033–0.17, 0–1.32]	0.058 [0.03–0.14, 0–1.32]	0.072 [0.04–0.18, 0.01–0.55]	0.058 [0.03–0.14, 0–1.32]	0.064 [0.03–0.15, 0–1.19]
Age in years	35.0 [27.3–40.8, 19.1–57.8]	34.5 [27.0–39.8, 19.1–55.6]	36.8 [29.9–42.4, 19.6–57.8]	33.2 [27.3–38.4, 19.1–55.6]	35.8 [27.0–41.2, 19.4–57.8]
EDSS	1.5 [1.0–2.0, 0–8.5]	1.0 [1.0–2.0, 0–4.0]	2.0 [1.5–2.5, 0–8.5]	1.5 [1.0–2.0, 0–3.0]	1.5 [1.0–2.0, 0–8.5]

Values are given in median [interquartile range, minimum–maximum]

*CIS* clinically isolated syndrome, *EDSS* score on the expanded disability status scale, *RRMS* relapsing–remitting multiple sclerosis, *SC* spinal cord, *tSSEP* tibial nerve somatosensory evoked potentials

**Table 2:** Comparison of subgroups

	Subgroup 1 vs 2 *p* value	Subgroup 1 tSSEP & SC MRI normal	Subgroup 2 tSSEP/SC MRI normal/abnormal	Subgroup 3 tSSEP & SC MRI abnormal	Subgroup 2 vs 3 *p* value
*n*		48	78	32	
Age (years)	0.8	33.7 [27.5–38.2]	35.0 [26.4–40.5]	37.0 [30.5–42.3]	0.1
Female/male	1.0	34/14	55/23	21/11	0.6
EDSS	0.3	1.5 [1.0–2.0)	1.0 [0–2.0]	2.0 [1.5–2.5]	< 0.001
Disease duration (years)	0.7	0.057 [0.03–0.16]	0.058 [0.03–1.39]	0.071 [0.04–0.18]	0.2
P40 latency (ms/m)	0.7	23.8 [23.1–24.4]	23.9 [23.1–24.6]	27.5 [26.5–30.3]*	n/a
SC lesion V (µL)	n/a	0	100 [59.4–343]	398 [189–663]	< 0.001

Values are given in median [interquartile range]. Apart from the comparison of the female/male ratios (Fisher’s exact test), Wilcoxon–Mann–Whitney-Test was used for group comparisons. EDSS, score on the expanded disability status scale; SC lesion V, spinal cord lesion volume. *If only one P40 value was measurable, this one was taken, whereas the 15 patients with no P40 latency measurable were excluded from this calculation

## Results

### Study participants and overview of findings

**Fig. 1:**
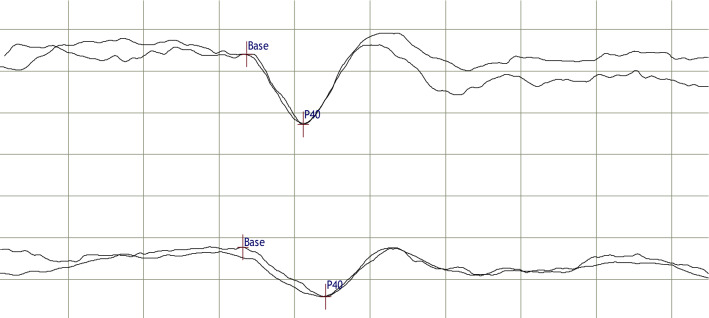
Tibial nerve somatosensory evoked potentials of the patient with increased P40 latency but without a corresponding lesion on brain or spinal cord magnetic resonance imaging. At the time of measurement, she was twenty-year-old, had optic neuritis, and was diagnosed with clinically isolated syndrome; she later developed multiple sclerosis. P40 latencies (corrected for the body height of 1.69 m)/amplitudes were 41.1 ms (24.3 ms/m)/3.4 µV after stimulation of the right tibial nerve (upper curve) and 44.1 ms (26.1 ms/m)/2.4 µV after stimulation of the left tibial nerve. Horizontal scaling, 10 ms/division; vertical scaling, 2 µV/division. Averaging was performed twice on each side with 185, and 186 (right) as well as 133, and 191 (left) stimuli, respectively

From our database, we identified a cohort of 173 patients with the complete dataset. In 12 of these patients, a relapse was reported in their medical records during the predefined interval of 200 days, leaving 161 patients for analysis. Demographic data are summarized in Table 1. Abnormal median nerve SSEP were rare (8 of 161). Likewise, lesions within the spinocortical pathway in the brain were rare and their inclusion in statistical models did not increase explained variance in a meaningful way. Only 3 patients had abnormal tSSEP but no lesion on SC MRI. One patient showed a symptomatic lesion in the right pontomedullary junction on brain MRI, well in accordance with the observed increased latency after stimulation of the left tibial nerve. Another patient with a symptomatic lesion in the posterior limb of the right internal capsule correspondingly showed a delayed latency of left tibial nerve SSEPs. However, one patient showed an increased latency slightly outside the normal range after stimulation of the left tibial nerve (Fig. 1), for whom we found an explanatory lesion neither on brain nor on SC MRI although coverage of the central nervous system was complete. Neither had this patient reported any symptoms suggestive of corticospinal pathway involvement. Against this backdrop, only analyses of SC MRI and tSSEP will be reported.

### Diagnostic sensitivity and association with disability

Results on diagnostic sensitivity are summarized in Table 1. SC MRI was more sensitive to detect SC involvement. Sixty-eight % (110 of 161) of the patients had an abnormal SC MRI but only 22% (35 of 161) of patients had abnormal tSSEP (*p* < 0.001), which were not measurable in 15 patients. Comparing scores on EDSS between patients without and with SC lesions did not yield significant differences (median, range 1.5, 0–3.0 vs 1.5, 0–8.5; *p* = 0.6). However, in the group of the 110 patients with lesions on SC MRI, SC lesion volume correlated with EDSS (Spearman rho 0.29, *p* = 0.002) even after correction for tSSEP (abnormal, 1; normal, 0; *p* = 0.044). Further, we compared the subgroup with normal and with abnormal tSSEP (i.e., subgroup 2 vs. 3, Table 2); abnormal tSSEP went along with higher SC lesion volumes and higher scores on EDSS. Patients with abnormal tSSEP had significantly higher scores on EDSS than patients with normal tSSEP (median, range 2.0, 0–8.5 vs. 1.0, 0–4.0, *p* = 0.001). In the group of 126 patients with normal tSSEP, we compared the subgroup without and with lesions on SC MRI (i.e., subgroup 1 vs 2, Table 2); we did neither observe a difference in disability (EDSS) nor in P40 latencies. Yet we found an association of P40 latency with EDSS (Spearman rho 0.17, *p* = 0.046). Of note, this correlation remained significant after controlling for SC lesions (abnormal, 1; normal, 0; *p* = 0.039).

## Discussion

To the best of our knowledge, this is the first study relating SC MRI with full axial coverage to SSEP data in early MS. In our cohort, SC MRI was more sensitive to SC involvement whereas tSSEP were more closely related to disability. We will discuss methodological issues, the underpinning of abnormal SSEP, and the value of both diagnostic measures as biomarkers [14]. We will also acknowledge limitations.

Our cohort was in a very early stage of MS with a median disease duration of one month and a maximum disease duration of 2 years. The significantly higher percentage of patients with abnormal SC MRI compared to tSSEP is well in accordance with the current diagnostic criteria of MS [2] incorporating only SC lesions detected by SC MRI but not abnormal SSEP. Yet we did not expect such a clear difference from the literature. While the percentage of patients with SC lesions (68%) was well in the range reported in the literature [15–18], the percentage of patients with abnormal tSSEP was low (22%). Other studies reported abnormal findings in > 80% [4, 6, 19] but had included patients more severely affected and in later stages. Because of the lack of a commonly accepted standard to quantify abnormalities for both tSSEP and SC MRI, we dichotomized our results (normal vs. abnormal) to compare sensitivity and performed correlation analyses in subgroups. The largest, in part overlapping, groups were these with abnormal SC MRI, and with normal tSSEP; both allowing for correlation analyses as, by definition, lesion volume from SC MRI was > 0 and P40 latency measurable. We also performed subgroup analyses in a fully parallel manner to treat both parameters equally.

In early studies [20, 21], the substrate of SSEP changes was termed ‘lesion’ [1]. Yet it has remained unclear to what degree this is visible on conventional T2-weighted MRI. Of note, studies using visually evoked potentials demonstrated abnormal findings in the absence of overt acute inflammatory activity (i.e. a history of previous optic neuritis) suggesting the possibility of further mechanisms leading to abnormal EP [22]. However, our results are largely compatible with the ‘lesion’ hypothesis. In 161 patients, we only observed a single case with abnormal tSSEP in the absence of a corresponding lesion on MRI along the spinocortical pathway; in this single case, P40 latency was only slightly outside the normal range so that it remains open whether this finding is related to MS at all. More interestingly, our results also provide evidence that tSSEP contain hidden information in the normal range as we here observed a remarkable correlation with disability (EDSS) even surviving correction for the existence of SC lesions. This association may be of value for correlation analyses in large groups. Nevertheless, we do not see a realistic way to leverage this information in clinical routine (i.e., at the individual level).

Regarding the demonstration of SC changes to aid the diagnosis of MS, our results are clear. SC MRI was more sensitive in detecting SC changes and is, hence, the more sensitive diagnostic biomarker—in this respect attributing a meaning to tSSEP only in case of an unavailable SC MRI. However, tSSEP were more closely associated with disability than SC MRI. In addition, our analyses of groups and subgroups suggested complementary information of both methods. These findings deserve recapitulation. tSSEP correlated with disability even in the normal range. When dichotomizing results, tSSEP but not SC MRI was able to discern more from less disabled patients. In the group of patients with SC lesions, lesion volume correlated with disability but, of note, the subgroup with abnormal tSSEP still showed more disability than the subgroup with normal tSSEP. A simple and straight forward explanation for these findings is that of a threshold effect with SC MRI having the lower threshold for being classified as abnormal. Compatible with our findings, this should lead to more patients being classified as abnormal by the methods with the lower threshold. As only one (small asymptomatic) lesion suffices to be classified as abnormal SC MRI, abnormal findings in this low-threshold test are more likely to go along with little or no disability than do abnormal findings in the high-threshold test (i.e., tSSEP in our case). Again, compatible with our findings, differences in disability between patients classified as abnormal and normal may be higher in the high-threshold method. However, even in statistical models comprising both methods, abnormal tSSEP were still (i.e., independently) related to more severe disability. The latter finding cannot merely be explained by a threshold effect. Differences in lesions may not be visible on MRI but have effects on SSEP measures such as eloquence of location (i.e., lesion volume within the spinocortical projection), and destructiveness (e.g., degree of demyelination, axonal loss, and remyelination). Our results are compatible with these mechanisms but unable to differentiate further. Moreover, it should be kept in mind that prognostication by measures such as MRI-based lesion volume or electrophysiological latencies likely results from autocorrelation, meaning that patients with higher values of a measure at baseline will also show a stronger increase of this measure in the later course. In conclusion, our results (of a closer relation of tSSEP to disability compared to SC MRI) are compatible with a potential value of tSSEP as a prognostic biomarker in complementation of SC MRI. Last but not least, currently available evidence on the prognostic value of evoked potentials seems more robust [5–9] than that of lesions detected by SC MRI [16–18, 23–25] although we are not aware of a study having directly compared both methods in this respect.

We acknowledge the limitations of our study. The number of patients is unlikely to have been large enough to cover the heterogeneity of mechanisms by which lesions can cause symptoms. Tibial SSEP cover SC pathways only incompletely and it is likely a matter of cohort size to find patients with SC lesions not changing tSSEP but causing severe clinical deficits. Moreover, our conclusions may not apply to later stages.

In summary, our results point to SC lesions, as visible on T2-weighed MRI, as the main driver of abnormal findings of SSEP in early MS. Accordingly, whole SC MRI is the more sensitive diagnostic biomarker than tSSEP. However, as changes in tSSEP were more closely related to disability, our data are, in principle, compatible with a potential role of tSSEP as a prognostic biomarker in the complementation of MRI, which however necessitates direct investigation.
